# Effect of Hydroxychloroquine on Proteinuria and Inflammatory Markers in Chronic Glomerulopathy: A Proof-of-Concept Randomized Controlled Trial (HCQ-CGN Trial)

**DOI:** 10.1159/gdz/acyag005

**Published:** 2026-09-29

**Authors:** Thitirat Supasil, Paramat Thimachai, Ouppatham Supasyndh, Bancha Satirapoj

**Affiliations:** Division of Nephrology, Department of Medicine, Phramongkutklao Hospital and College of Medicine, Bangkok, Thailand

**Keywords:** Chronic glomerulopathy, Hydroxychloroquine, Proteinuria, Inflammatory markers, Glomerulonephropathy

## Abstract

**Introduction:**

Hydroxychloroquine (HCQ) has immunomodulatory and anti-inflammatory properties that may provide additional benefit in chronic proteinuric glomerulopathy (CGN). This study aimed to evaluate the short-term effects of adjunctive HCQ on proteinuria and inflammatory biomarkers in patients with stable CGN receiving optimized supportive therapy.

**Methods:**

In this single-center, double-blind, placebo-controlled proof-of-concept randomized trial, 43 patients with CGN (urine protein-to-creatinine ratio of ≥0.5 g/g and estimated glomerular filtration rate [eGFR] of >20 mL/min/1.73 m^2^) were randomized to receive eGFR-adjusted HCQ (*n* = 21) or placebo (*n* = 22) for 4 months. The primary outcome was change in proteinuria; secondary outcomes included interleukin-6 (IL-6), high-sensitivity C-reactive protein (hsCRP), eGFR, and safety.

**Results:**

After 4 months, the median percentage change in proteinuria was −27.9% (IQR, −55.1 to −15.5) with HCQ versus +20.7% (IQR, −6.9 to +45.4) with placebo. In mixed-effects maximum-likelihood regression adjusted for systolic blood pressure, HCQ was associated with a greater reduction in proteinuria than placebo (β, −118.55; 95% CI, −208.07 to −29.03; *p* = 0.009). A ≥30% reduction in proteinuria occurred more frequently with HCQ than placebo (50.0% vs. 14.3%; *p* = 0.014). IL-6 and hsCRP were also reduced with HCQ. No significant between-group difference in eGFR or serious adverse events was observed.

**Conclusion:**

Adjunctive HCQ was associated with a short-term reduction in proteinuria in patients with stable chronic proteinuric glomerulopathy receiving optimized supportive therapy. Exploratory reductions in IL-6 and hsCRP were also observed. These proof-of-concept findings support further evaluation of HCQ in larger, longer, biopsy-based, disease-specific randomized trials.

## Introduction

Hydroxychloroquine (HCQ), an antimalarial drug, has been widely used because of its safety profile and tolerability. It is recognized for its immunomodulatory and anti-inflammatory properties in the management of rheumatological disorders such as systemic lupus erythematosus and rheumatoid arthritis [1]. Its advantages in lupus nephritis (LN) include a reduction in renal disease exacerbation, enhanced renal remission rates, and extended duration until end-stage kidney disease occurs [2]. Despite decades of clinical application of HCQ, its exact mechanism of action remains unclear. The efficacy of HCQ may result from its ability to inhibit various cascades of the inflammatory response, such as its inhibitory effect on autoimmunity, prevention of cellular autophagy, inhibition of Toll-like receptor activation, and decrease in the production of inflammatory cytokines. HCQ is being utilized for several medical problems beyond its original purpose, since recent studies indicate a reduction in proteinuria in IgA nephropathy (IgAN) and primary membranous nephropathy and its impact on atherosclerosis [3].

The KDIGO 2021 (Kidney Disease Improving Global Outcomes, KDIGO) guidelines for LN advocate the administration of HCQ or alternative antimalarial agents in patients with kidney disease, provided there are no contraindications, with a grade 1C recommendation level [4]. A randomized controlled trial involving Chinese patients with IgAN compared HCQ treatment to placebo, revealing that those administered HCQ for 6 months had a statistically significant reduction in proteinuria relative to the placebo group [5].

Chronic glomerulopathy (CGN), along with chronic glomerulonephritis and diabetic nephropathy (DN), contributes to chronic kidney disease (CKD), which poses a substantial public health challenge resulting in diminished quality of life, elevated mortality rates, and complications associated with renal and cardiovascular diseases [6]. Studies indicate that CKD affects over 10% of the global population [7, 8] and between 4.3% and 13.8% of the Thai population [9], with a risk of advancing to end-stage kidney disease if not properly managed. Data from kidney biopsies reveal that the predominant causes of glomerulopathy in the Thai population are LN, IgAN, and DN, respectively [10]. In addition to the reduction in the glomerular filtration rate and elevated proteinuria, which are significant indications of deteriorating kidney function, chronic inflammation has been recognized as a mechanism that contributes to increased mortality rates. The large-scale Chronic Renal Insufficiency Cohort (CRIC) study identified higher concentrations of inflammatory markers in circulation, including interleukin-1 (IL-1), interleukin-6 (IL-6), TNF-alpha, high-sensitivity C-reactive protein (hsCRP), and fibrinogen, in patients exhibiting diminished glomerular filtration rate and elevated proteinuria [11].

This study aimed to examine the short-term impact of HCQ on proteinuria and inflammatory markers in individuals with CGN. Rather than targeting a single disease-specific mechanism, this proof-of-concept trial was designed to probe a common clinical pathway – proteinuria and systemic inflammation – shared across proteinuric chronic glomerulopathies. This is the first randomized controlled trial conducted on Thai patients with CGN and is intended to generate preliminary signals to justify larger, longer, and disease-specific trials of HCQ as an adjuvant treatment for CGN in addition to standard therapy.

## Methods

### Study Designs

This study was a single-center, hospital-based, double-blind, randomized, placebo-controlled proof-of-concept trial that enrolled patients with CGN at the Phramongkutklao Hospital, Bangkok, Thailand. A block of four randomizations randomly assigned patients, stratified by disease, to either oral HCQ or placebo in patients with CGN receiving maximal supportive treatment according to the KDIGO glomerulonephritis guidelines [4]. This study was conducted in accordance with the principles of the Declaration of Helsinki and reported in accordance with the Consolidated Standards of Reporting Trials (CONSORT) guidelines (online supplementary material; for all online suppl. material, see https://doi.org/10.1159/gdz/acyag005). All participants provided written informed consent before enrollment in the study. This trial was registered under the Thai Clinical Trials code TCTR20250319019.

### Study Populations

The main study inclusion criteria were age over 20 years, a diagnosis of CGN from any cause with or without biopsy-proven CGN (distribution of etiologies of CGN as described in online suppl. Table 1), estimated glomerular filtration rate (eGFR) of >20 mL/min/1.73 m^2^ (calculated using the Chronic Kidney Disease Epidemiology Collaboration [CKD-EPI] creatinine equation), and protein excretion of at least 0.50 grams per day despite receiving a maximum tolerable dose of renin-angiotensin-aldosterone system (RAAS) inhibitor for at least 12 weeks (3 months). To ensure that background therapy was stable and would not confound the proteinuria endpoint, all participants were required to be on a stable, optimized dose of RAAS inhibitor (ACEi or ARB) for at least 12 weeks prior to enrollment, with no dose changes permitted during the 4-month study period, and no new SGLT2 inhibitor or mineralocorticoid receptor antagonist (MRA) was initiated during the study. The exclusion criteria were the use of systemic immunosuppressive therapy except for maintenance low-dose steroids (prednisolone of ≤10 mg/day, stable for ≥12 weeks) at the time of enrollment, an indication for a new regimen of corticosteroids (crescentic IgAN or minimal change disease with IgA deposits), contraindications for HCQ therapy, severe and uncontrolled comorbid diseases, and current or planned pregnancy or lactation.

Because biopsy is the gold standard for histologic diagnosis but may be contraindicated or declined once CKD is established, we adopted pragmatic inclusion criteria permitting both biopsy-proven and clinically diagnosed CGN, reflecting real-world clinical practice at our center. The distribution of biopsy-proven versus clinically diagnosed participants is reported in Table 1, and a sensitivity analysis comparing these subgroups is presented in the Results.

**Table 1.** Baseline characteristics of the HCQ group and placebo group

Baseline characteristics	HCQ group (*n* = 21)	Placebo group (*n* = 22)	Total (*n* = 43)
Age, years	54.90±15.29	48.41±16.65	51.58±16.15
Male sex	12 (57.1%)	11 (50%)	23 (53.5%)
Period from biopsy to randomization, months	31.73±40.93	20.5±21.89	27.24±34.48
Presence of renal tissue pathology	15 (71.1%)	10 (45.5%)	25 (58.1%)
Systolic BP, mm Hg	133.52±5.44	126.09±8.73	129.72±8.15
Diastolic BP, mm Hg	77.71±9.72	73.45±6.54	75.53±8.43
Serum Cr, mg/dL	1.58±0.67	1.60±0.80	1.59±0.73
Baseline eGFR, mL/min/1.73 m^2^	55.06±29.46	54.48±24.84	54.76±26.87
Serum albumin, g/L	4.22±0.37	4.21±0.24	4.22±0.31
Baseline proteinuria – UPCR, g/g	1.53±0.94	1.09±0.85	1.3±0.91
Baseline hsCRP, mg/L	2.73±4.22	1.8±1.9	2.26±3.27
Median (IQR)	1.03 (0.47, 3.36)	0.92 (0.48, 2.44)	1.03 (0.47, 2.51)
Baseline IL-6, pg/mL	3.85±4.02	2.26±1.76	3.04±3.15
Median (IQR)	3.31 (1.79, 4.28)	1.56 (0.95, 3.38)	2.37 (1.11, 3.65)
Etiologies of CGN			
IgA nephropathy (IgAN)	7 (33.3%)	7 (31.8%)	14 (32.6%)
Diabetic nephropathy (DN) or suspected DN	5 (23.8%)	6 (27.2%)	11 (25.6%)
Other	9 (42.9%)	9 (40.9%)	18 (41.9%)
Therapy with RAASi	20 (95.2%)	20 (90.9%)	40 (93%)
ACEi	4 (19%)	4 (18.2%)	8 (18.6%)
ARB	16 (76.2%)	16 (72.7%)	32 (74.4%)
% of maximum labeled dose	10 (50%)	8 (40%)	18 (41.9%)
Therapy with SGLT2i	6 (28.6%)	6 (27.3%)	12 (27.9%)
Therapy with nsMRA	1 (4.8%)	0	1 (2.3%)
Therapy with GLP-1 RAs	2 (9.5%)	0	2 (4.7%)
Low-dose prednisolone	2 (9.5%)	3 (13.6%)	5 (11.6%)
Previous corticosteroid/immunosuppressive therapy	5 (23.8%)	5 (22.7%)	10 (23.3%)

Values are presented as mean ± standard deviation or median (IQR) for continuous variables, and n (%) for categorical variables. *p* values were calculated using the independent *t* test or the Mann-Whitney U test for continuous variables, and the chi-square test for categorical variables. Statistical significance was defined as *p* < 0.05.

ACEi, angiotensin-converting enzyme inhibitor; ARB, angiotensin II receptor blocker; BP, blood pressure; CGN, chronic glomerulonephritis; DN, diabetic nephropathy; eGFR, estimated glomerular filtration rate; GLP-1 RAs, glucagon-like peptide-1 receptor agonists; HCQ, hydroxychloroquine; hsCRP, high-sensitivity C-reactive protein; IgAN, IgA nephropathy; IL-6, interleukin-6, nsMRA, nonsteroidal mineralocorticoid receptor antagonists; RAASi, renin-angiotensin-aldosterone system inhibitor; Scr, serum creatinine; SGLT2i, sodium-glucose cotransporter-2 inhibitors; UPCR, urine protein-to-creatinine ratio.

### Randomization and Masking

Patients were randomly assigned 1:1 to receive oral HCQ (hydroxychloroquine sulfate tablets; Hydroquin, provided by Sun Pharmaceutical Industries Ltd.) or a placebo using a block of four randomizations stratified by disease (IgAN, DN, and others) with two HCQ treatments and two controlled allocations per block, which aimed to balance the number of participants between the HCQ and control groups. HCQ and the matched placebo were uniformly packaged and numbered by the independent institute, Pharmaceutical Intellectual Center “Prachote Plengwittaya,” Faculty of Pharmacy, Silpakorn University, Sanam Chandra Palace Campus. The study medication was dispensed and provided to the trial participants by an investigator (nephrologists and nephrology fellows). The investigators and participants were unaware of the code until after the end of the study or until serious adverse events (SAEs) occurred. Medication reminders were provided to the participants by phone every 2 weeks. At least 80% of the prescribed treatment dose was considered good adherence. The patients and investigators were blinded to the treatment assignments for the duration of the study.

### Treatment and Follow-Up

The patients received their assigned treatment for 4 months. The HCQ dose was adjusted according to baseline eGFR to reduce the risk of drug accumulation in impaired renal function: 200 mg orally (1 tablet) twice daily for patients with eGFR of >60 mL/min/1.73 m^2^, 300 mg orally (1.5 tablets) once daily for patients with eGFR between 45 and 59 mL/min/1.73 m^2^, 200 mg orally (1 tablet) once daily for patients with eGFR between 30 and 44 mL/min/1.73 m^2^, and 100 mg (0.5 tablet) once daily for patients with eGFR between 20 and 29 mL/min/1.73 m^2^. Treatment was discontinued in participants with a >30% decline in eGFR. No new treatment was administered during the study. All patients followed a standardized hypertension management protocol targeting a blood pressure of <130/80 mm Hg and received uniform low-sodium dietary counseling at each visit; this protocol was applied equally to both arms. Patients were assessed at baseline and at 2 and 4 months after the study. Assessments included adherence to the study medicines, blood pressure, adverse event reporting, routine hematology, routine urinalysis, serum chemistry, morning urine protein-to-creatine ratio (UPCR), serum hsCRP, and serum IL-6. eGFR was calculated using the CKD-EPI equation using serum creatinine level. Fundus examinations were performed at baseline and at the last visit. A baseline 12-lead electrocardiogram (ECG) was obtained in all participants; continuous Holter monitoring and on-treatment follow-up ECG were not performed.

### Laboratory Measurement

IL-6 and hsCRP concentrations in the serum were determined at baseline and 4 months, as previously described. Serum IL-6 was evaluated using an in vitro chemiluminescence immunoassay kit for the quantitative determination of IL-6 in human serum and plasma using a MAGLUMI 600 series fully automated chemiluminescence immunoassay analyzer (Shenzhen New Industries Biomedical Engineering Co., Ltd. [Snibe]). Serum hsCRP was evaluated using an in vitro test for the quantitative determination of hsCRP in human plasma using the COBAS c and COBAS INTEGRA systems (Roche Diagnostics Co., Ltd.). Protein excretion was calculated using the first-morning UPCR.

### Outcomes

The primary outcome was the change in proteinuria (UPCR) from baseline to 4 months. The secondary and exploratory outcomes included the change in inflammatory markers (IL-6 and hsCRP) from baseline to 4 months, the frequency of patients achieving a 30% reduction in proteinuria, and the percentage change in eGFR. Only the primary proteinuria endpoint was formally prespecified for confirmatory inference; the biomarker and subgroup analyses were preplanned but exploratory and hypothesis-generating.

### Adverse Events and Safety

Predefined safety outcomes were total SAEs, serious allergies, ophthalmologic events, serious gastrointestinal symptoms, dermatologic changes, neuromuscular symptoms, cardiovascular or respiratory disorders, leukopenia, agranulocytosis, and aplastic anemia requiring hospitalization. Adverse events were ascertained systematically at each scheduled visit using a standardized checklist and open questioning. SAEs were defined according to the International Conference on Harmonization Guidelines for Clinical Safety Data Management.

### Statistical Analysis

Based on data from Liu et al. [5], if the mean ± standard deviation of the percentage change of proteinuria in the HCQ group was −38 ± 26.5 and that of the placebo group was −3.5 ± 29.2, a sample of 14 participants per arm would provide 90% power to detect the difference between groups, with a type I error rate of 0.05. Assuming a 20% dropout rate, we planned to recruit 36 participants (18 per group) for this study. All analyses were conducted in accordance with the intention-to-treat principle. Normally distributed data are presented as the mean ± standard deviation, and non-normally distributed data are presented as medians with interquartile ranges (IQRs). Categorical data are summarized as counts and percentages. The chi-squared test or Fisher’s exact test was used for categorical variables. For the primary proteinuria endpoint, both unadjusted and adjusted analyses are reported. The unadjusted between-group comparison used the Mann-Whitney U test on the percentage change in UPCR. The main adjusted analysis used mixed-effects maximum-likelihood (ML) regression with treatment group as the fixed effect and systolic blood pressure (SBP) as a covariate to address the baseline imbalance in SBP and to make full use of the repeated measures; the treatment effect is reported as a β coefficient with 95% confidence interval. Because UPCR and the inflammatory markers were right-skewed, a log mixed-effects ML regression model (on the log-transformed outcomes) was additionally fitted as a sensitivity analysis and yielded consistent results. The same mixed-effects ML regression approach, adjusted for SBP, was applied to the secondary endpoints (percentage and absolute change in hsCRP, IL-6, and eGFR) and to the prespecified etiology subgroups (IgAN, DN, and other). The independent influence of treatment, SBP, and baseline IL-6 on the change in proteinuria was further examined in unadjusted and adjusted mixed-effects models (online suppl. Table 2). Differences between groups were analyzed using mixed-effects ML regression. Differences within groups were analyzed using the dependent *t* test or the Wilcoxon signed-ranks test for continuous data. The frequency of patients with a 30% decrease in proteinuria at each time point was evaluated using a simple proportion calculation. Given the exploratory nature of the secondary and subgroup analyses in this small cohort, no formal multiplicity adjustment (gatekeeping hierarchy) was applied, and these results are interpreted as hypothesis-generating. Statistical significance was defined as a *p* value <0.05. Statistical analyses were performed using SPSS, version 30.

## Results

### Baseline Characteristics

From April 2024 to January 2025, a total of 169 eligible patients were screened, of whom 43 were eligible for the study and underwent randomization (21 in the HCQ group and 22 in the placebo group), as illustrated in Figure 1. One patient in the HCQ group discontinued the intervention based on the patient’s decision, and 1 patient in the placebo group was admitted for pneumonia; all 43 randomized participants were included in the intention-to-treat analysis (21 HCQ and 22 placebo). The dose of the investigational medication was not changed during the study. Adherence was high in both arms, and no participant fell below the 80% adherence threshold; therefore, no per-protocol exclusions for nonadherence were required. The 2 randomized groups had similar baseline characteristics with respect to age, sex, eGFR, serum creatinine, serum albumin, etiology, and background therapy (including RAAS inhibitor, SGLT2 inhibitor, nsMRA, and previous corticosteroid/immunosuppressive use), except that SBP in the HCQ group was slightly higher than that in the placebo group (133.5 ± 5.4 vs. 126.1 ± 8.7 mm Hg; *p* = 0.002), but stable SBP was observed during the study. Baseline etiology, renal-pathology status, diabetes status, and background medications stratified by arm are shown in Table 1, with the detailed distribution of renal pathology provided in online supplementary Table 1. The mean or median for the entire cohort baseline characteristics were as follows: proteinuria, UPCR, 1.3 ± 0.91 g/g; eGFR, 54.76 ± 26.87 mL/min/1.73 m^2^; IL-6, 2.37 (1.11, 3.65) pg/mL; and hsCRP, 1.03 (0.47, 2.51) mg/L. The main demographic data are presented in Table 1.

**Fig. 1.**
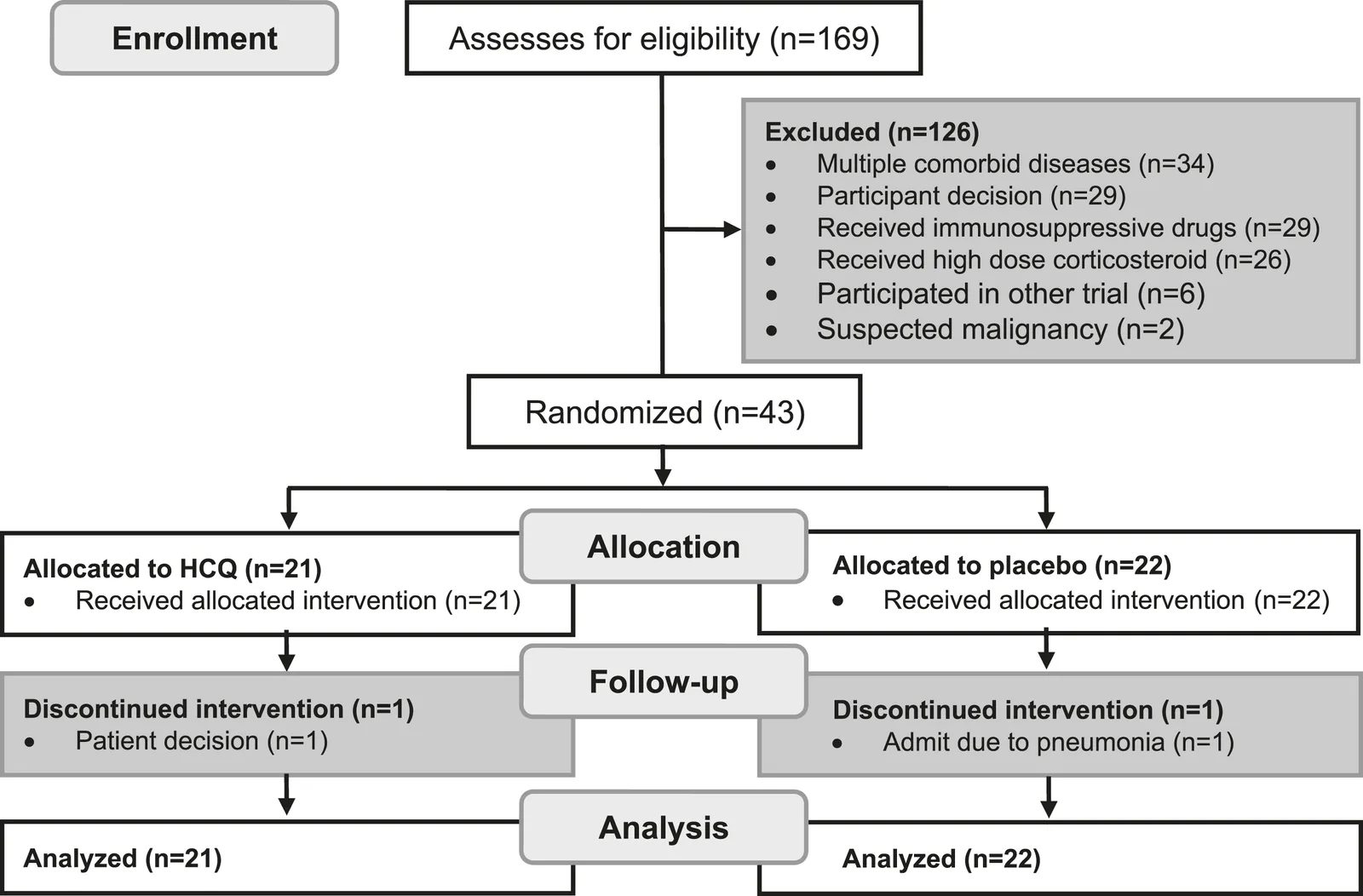
Flowchart of the study.

### Primary Endpoints

In the primary outcome analysis, proteinuria decreased in the HCQ group (median UPCR 1.53 g/g at baseline to 0.82 [IQR 0.55, 1.36] g/g at 4 months), whereas it increased in the placebo group (1.09 to 1.21 [0.67, 1.84] g/g). The median percentage change in UPCR from baseline to 4 months was −27.85% (IQR −55.10, −15.47) in the HCQ group vs. +20.71% (−6.91, +45.35) in the placebo group, and the median absolute change was −0.37 (−0.84, −0.18) g/g vs. +0.15 (−0.04, +0.39) g/g, respectively, as illustrated in Figure 2. In mixed-effects ML regression adjusted for SBP, HCQ was associated with a significantly greater reduction in proteinuria, whether expressed as the percentage change (β −118.55 [95% CI −208.07, −29.03], *p* = 0.009), the absolute change in g/g (β −1.01 [−1.64, −0.38], *p* = 0.002), or the month-4 proteinuria level (β −1.02 [−1.65, −0.40], *p* = 0.001). The unadjusted and adjusted estimates are presented in Table 2. A sensitivity analysis using a log mixed-effects ML regression model on the log-transformed UPCR was concordant with the main analysis: HCQ was associated with a significantly greater reduction in proteinuria (adjusted ratio of geometric means for the between-group difference in change 0.51 [95% CI 0.36, 0.72], *p* < 0.001), corresponding to an approximately 49% lower proteinuria in the HCQ group relative to placebo and confirming the robustness of the treatment effect to the choice of model (online suppl. Table 3). A prespecified exploratory subgroup analysis was defined by causes of CGN: IgAN, DN, or other. The reduction in proteinuria favored HCQ in IgAN (percentage change −30.16% vs. +20.71%; adjusted β −67.04 [−127.89, −6.19], *p* = 0.031) and in other causes (−32.22% vs. +34.26%; adjusted β −270.82 [−483.59, −58.05], *p* = 0.013), and in the combined non-DN group (IgAN + other; adjusted β for percentage change −190.49 [−315.82, −65.16], *p* = 0.003). In the DN subgroup, the change favored HCQ in direction (−22.94% vs. +0.71%) but was not statistically significant (adjusted β 11.32 [−63.43, 86.06], *p* = 0.767; Table 2). A sensitivity analysis by histologic status was consistent in direction, with reductions in proteinuria observed in both biopsy-proven and clinically diagnosed participants.

**Fig. 2.**
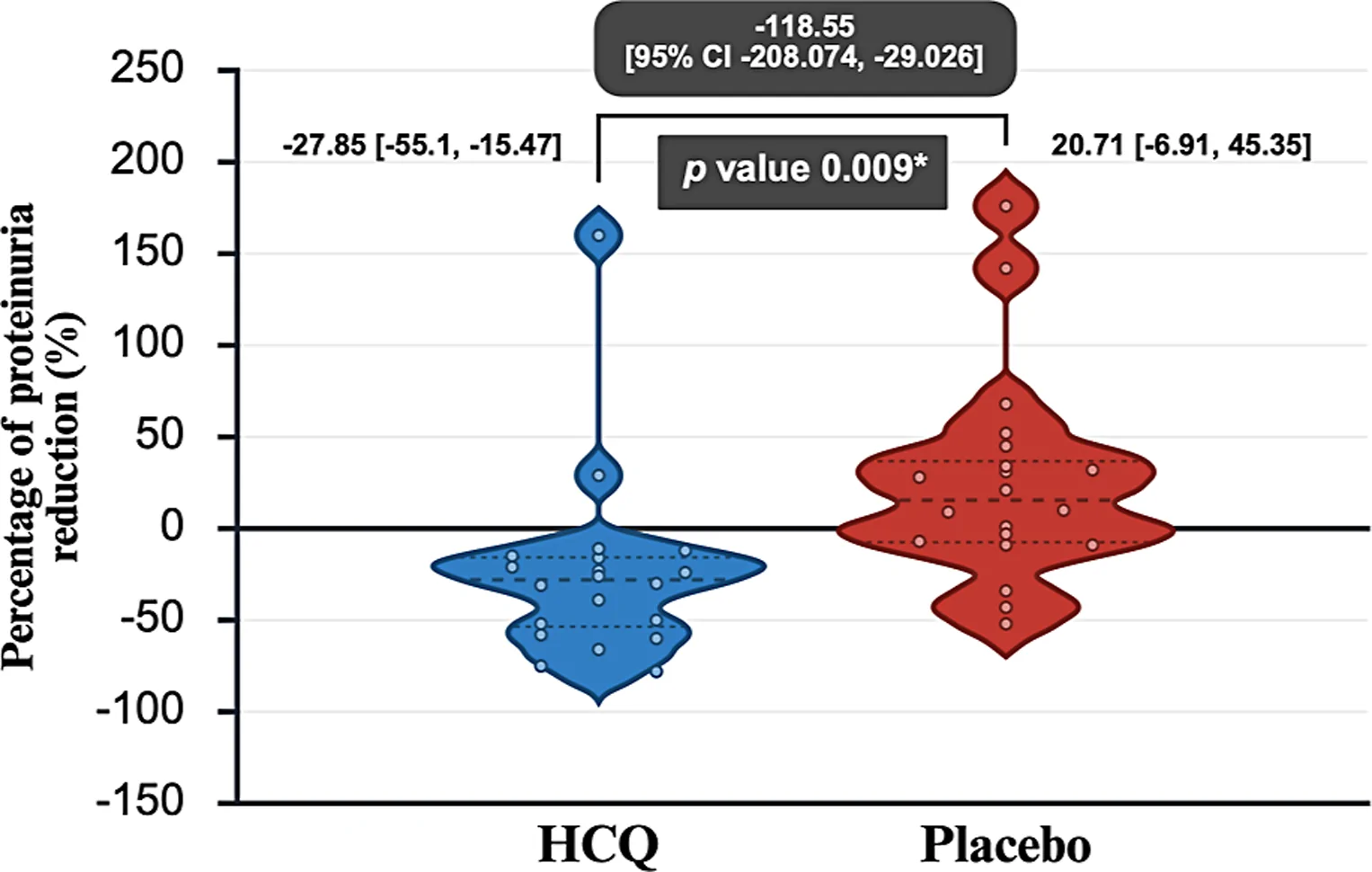
Effects of HCQ add-on therapy with 4 months of follow-up. In the primary outcome analysis, proteinuria decreased in the HCQ group (median UPCR 1.53 g/g at baseline to 0.82 [IQR 0.55, 1.36] g/g at 4 months), whereas it increased in the placebo group (1.09 to 1.21 [0.67, 1.84] g/g). The median percentage change in UPCR from baseline to 4 months was −27.85% (IQR −55.10, −15.47) in the HCQ group vs. +20.71% (−6.91, +45.35) in the placebo group, and the median absolute change was −0.37 (−0.84, −0.18) g/g vs. +0.15 (−0.04, +0.39) g/g, respectively.

**Table 2.** Effects of HCQ on primary outcomes, median (IQR)

Primary outcome	HCQ group (*n* = 21)	Placebo group (*n* = 22)	Beta (95% CI)	*p* value
Overall CGN, *n*	21	22	​	​
Change in proteinuria at 4 m, %	−27.85 (−55.10, −15.47)	20.71 (−6.91, 45.35)	−118.55 (−208.07, −29.03)	0.009*
Change in proteinuria at 4 m, g/g	−0.37 (−0.84, −0.18)	0.15 (−0.04, 0.39)	−1.01 (−1.64, −0.38)	0.002*
Proteinuria at 4 m, g/g	0.82 (0.55, 1.36)	1.21 (0.67, 1.84)	−1.02 (−1.65, −0.40)	0.001*
IgA nephropathy, *n*	7	7	​	​
Change in proteinuria at 4 m, %	−30.16 (−65.56, −21.09)	20.71 (−42.82, 32.21)	−67.04 (−127.89, −6.19)	0.031*
Change in proteinuria at 4 m, g/g	−0.35 (−0.94, −0.20)	0.11 (−0.35, 0.34)	−0.57 (−1.01, −0.13)	0.011*
Proteinuria at 4 m, g/g	0.63 (0.37, 0.68)	0.67 (0.42, 1.26)	−0.54 (−0.95, −0.13)	0.010*
Diabetic nephropathy, *n*	5	6	​	​
Change in proteinuria at 4 m, %	−22.94 (−31.26, 29.08)	0.71 (−6.91, 9.63)	11.32 (−63.43, 86.06)	0.767
Change in proteinuria at 4 m, g/g	−0.19 (−0.67, 0.55)	0.01 (−0.05, 0.24)	0.27 (−1.07, 1.60)	0.697
Proteinuria at 4 m, g/g	1.47 (0.75, 2.43)	2.32 (1.74, 2.74)	0.12 (−1.27, 1.50)	0.871
Other, *n*	9	9	​	​
Change in proteinuria at 4 m, %	−32.22 (−55.10, −15.47)	34.26 (9.28, 68.10)	−270.82 (−483.59, −58.05)	0.013*
Change in proteinuria at 4 m, g/g	−0.57 (−1.31, −0.20)	0.37 (0.10, 0.43)	−1.99 (−3.18, −0.80)	0.001*
Proteinuria at 4 m, g/g	0.97 (0.67, 1.27)	1.21 (0.96, 1.84)	−1.98 (−3.16, −0.81)	0.001*
Other than DN (IgAN + Other)	16	16	​	​
Change in proteinuria at 4 m, %	−30.16 (−59.59, −16.00)	29.53 (−5.87, 59.96)	−190.49 (−315.82, −65.16)	0.003*
Change in proteinuria at 4 m, g/g	−0.40 (−1.20, −0.20)	0.24 (−0.03, 0.41)	−1.43 (−2.16, −0.71)	<0.001*
Proteinuria at 4 m, g/g	0.68 (0.45, 1.15)	1.02 (0.58, 1.32)	−1.42 (−2.14, −0.71)	<0.001*

Values are presented as beta coefficients with 95% confidence intervals after adjusting for SBP. *p* values were calculated using mixed-effects maximum likelihood (ML) regression. Statistical significance was defined as *p* < 0.05.

### Secondary and Exploratory Endpoints

The inflammatory-marker analyses were preplanned secondary endpoints and are detailed in Table 3. For serum IL-6, the median percentage change was −27.65% (IQR −48.56, −5.90) in the HCQ group vs. +47.98% (17.80, 96.77) in the placebo group; in mixed-effects ML regression adjusted for SBP, this corresponded to an adjusted β of −80.42 (95% CI −120.18, −40.66), *p* < 0.001. The effect was most pronounced in the IgAN (adjusted β −105.87 [−155.97, −55.76], *p* < 0.001) and DN subgroups (−133.76 [−169.06, −98.46], *p* < 0.001). For hsCRP, the median percentage change was −31.85% (−60.48, −3.03) in the HCQ group vs. +1.23% (−27.5, 44.32) in the placebo group (adjusted β −52.59 [−89.47, −15.71], *p* = 0.005). No statistically significant differences were observed in the percentage change in the eGFR (adjusted β −3.85 [−14.71, 7.01], *p* = 0.487; Table 3), indicating preserved kidney function in both groups over the 4-month period. Although these secondary findings were statistically significant, they should be interpreted cautiously given the small sample, the short follow-up, the nonspecific nature of IL-6 and hsCRP, and the multiplicity of secondary testing. Of the 41 patients who completed the month 4 visit, 10 (50%) patients in the HCQ group showed a 30% decrease in proteinuria at 4 months compared with 3 (14.3%) patients in the placebo group (*p* = 0.014), as shown in Figure 3.

**Table 3.** Effects of HCQ on secondary outcomes, median (IQR)

Secondary outcome	HCQ group	Placebo group	Beta (95% CI)	*p* value
	median (IQR)	median (IQR)		
Change in hsCRP at 4 m, %				
Overall CGN	−31.85 (−60.48, −3.03)	1.23 (−27.5, 44.32)	−52.59 (−89.47, −15.71)	0.005*
IgA nephropathy	−56.73 (−85.36, −44.76)	10.00 (−4.48, 81.25)	−85.41 (−141.14, −29.67)	0.003*
Diabetic nephropathy	−20.87 (−29.75, −3.03)	34.81 (−24.53, 97.19)	−67.84 (−129.62, −6.07)	0.031*
Other	−10.18 (−45.82, 29.41)	−20.63 (−48.34, 39.13)	−0.90 (−67.51, 65.72)	0.979
Change in hsCRP at 4 m, mg/L				
Overall CGN	−0.32 (−1.33, −0.01)	0.03 (−0.12, 0.36)	−2.13 (−4.21, −0.06)	0.044*
IgA nephropathy	−1.60 (−5.28, −0.49)	0.05 (−0.09, 0.34)	−5.03 (−9.39, −0.66)	0.024*
Diabetic nephropathy	−0.43 (−1.00, −0.01)	0.30 (−1.20, 0.60)	−1.52 (−3.48, 0.44)	0.129
Other	−0.11 (−0.27, 0.12)	−0.12 (−1.60, 0.36)	1.94 (0.38, 3.49)	0.015*
hsCRP at 4 m, mg/L				
Overall CGN	1.10 (0.30, 2.00)	1.30 (0.60, 1.80)	−0.45 (−1.42, 0.52)	0.362
IgA nephropathy	1.00 (0.60, 2.30)	0.90 (0.20, 1.90)	−1.07 (−3.59, 1.46)	0.408
Diabetic nephropathy	1.50(1.10, 1.60)	1.60 (1.30, 2.00)	−0.11 (−0.85, 0.63)	0.768
Other	0.50 (0.10, 2.0)]	1.10 (0.50, 1.50)	−0.56 (−2.01, 0.89)	0.448
Change in IL-6 at 4 m, %				
Overall CGN	−27.65 (−48.56, −5.90)	47.98 (17.80, 96.77)	−80.42 (−120.18, −40.66)	<0.001*
IgA nephropathy	−48.35 (−66.45, −27.65)	25.83 (17.80, 87.95)	−105.87 (−155.97, −55.76)	<0.001*
Diabetic nephropathy	−18.82 (−38.67, −14.77)	98.19 (71.03, 117.57)	−133.76 (−169.06, −98.46)	<0.001*
Other	9.02 (−46.72, 48.92)	34.24 (−43.75, 82.26)	−24.29 (−112.19, 63.61)	0.588
Change in IL-6 at 4 m, pg/mL				
Overall CGN	−0.49 (−2.37, −0.20)	0.62 (0.09, 1.32)	−2.40 (−4.79, −0.01)	0.049*
IgA nephropathy	−1.96 (−2.37, −0.49)	0.47 (0.09, 0.82)	−3.03 (−4.66, −1.41)	<0.001*
Diabetic nephropathy	−0.49 (−2.54, −0.40)	1.39 (1.18, 1.61)	−5.86 (−12.77, 1.04)	0.096
Other	0.15 (−2.08, 0.70)	0.33 (−2.28, 0.82)	−0.59 (−3.31, 2.14)	0.673
IL-6 at 4 m, pg/mL				
Overall CGN	2.20 (1.73, 2.77)	2.49 (1.64, 3.31)	−0.10 (−1.77, −0.22)	0.011*
IgA nephropathy	1.74 (1.30, 2.20)	2.46 (1.47, 3.34)	−3.06 (−4.03, −2.09)	<0.001*
Diabetic nephropathy	2.85 (1.73, 3.14)	3.00 (2.61, 3.41)	−0.48 (−1.66, 0.70)	0.429
Other	2.47 (2.13, 2.77)	2.21 (1.57, 2.82)	−0.57 (−1.52, 0.38)	0.238
Change in eGFR at 4 m, %				
Overall CGN	−0.09 (−6.5, 5.78)	0.43 (−11.34, 6.79)	−3.85 (−14.71, 7.01)	0.487
IgA nephropathy	1.62 (−20.35, 5.40)	0.43 (−11.93, 18.81)	−5.82 (−31.59, 19.96)	0.658
Diabetic nephropathy	−2.71 (−9.91, 12.90)	2.39 (−6.64, 2.74)	1.06 (−14.30, 16.42)	0.893
Other	−0.09 (−2.63, 4.71)	0 (−11.34, 9.87)	−15.77 (−33.43, 1.88)	0.08
Change in eGFR at 4 m				
Overall CGN	−0.17 (−3.18, 2.48)	0.11 (−4.21, 3.74)	−3.78 (−9.79, 2.24)	0.219
IgA nephropathy	0.62 (−15.39, 2.3)	0.11 (−11.76, 9.89)	−6.92 (−20.87, 7.03)	0.331
Diabetic nephropathy	−0.78 (−3.26, 4.71)	0.83 (−3.09, 1.04)	−0.88 (−5.56, 3.80)	0.713
Other	−0.24 (−2.62, 1.70)	0 (−8.65, 4.49)	−8.10 (−19.48, 3.29)	0.163
eGFR at 4 m				
Overall CGN	45.45 (32.1, 83.78)	52.16 (37.84, 67.65)	9.48 (−7.070, 26.02)	0.262
IgA nephropathy	48.38 (38.80, 78.49)	58.65 (47.7, 104.00)	17.91 (−14.13, 49.94)	0.273
Diabetic nephropathy	34.56 (29.64, 41.21)	35.59 (33.11, 38.94)	2.020 (−4.30, 8.34)	0.531
Other	72.91 (37.57, 95.63)	58.83 (49.99, 68.92)]	4.23 (−20.41, 28.86)	0.737

Values are presented as beta coefficients with 95% confidence intervals after adjusting for SBP. *p* values were calculated using mixed-effects maximum likelihood (ML) regression. Statistical significance was defined as *p* < 0.05.

**Fig. 3.**
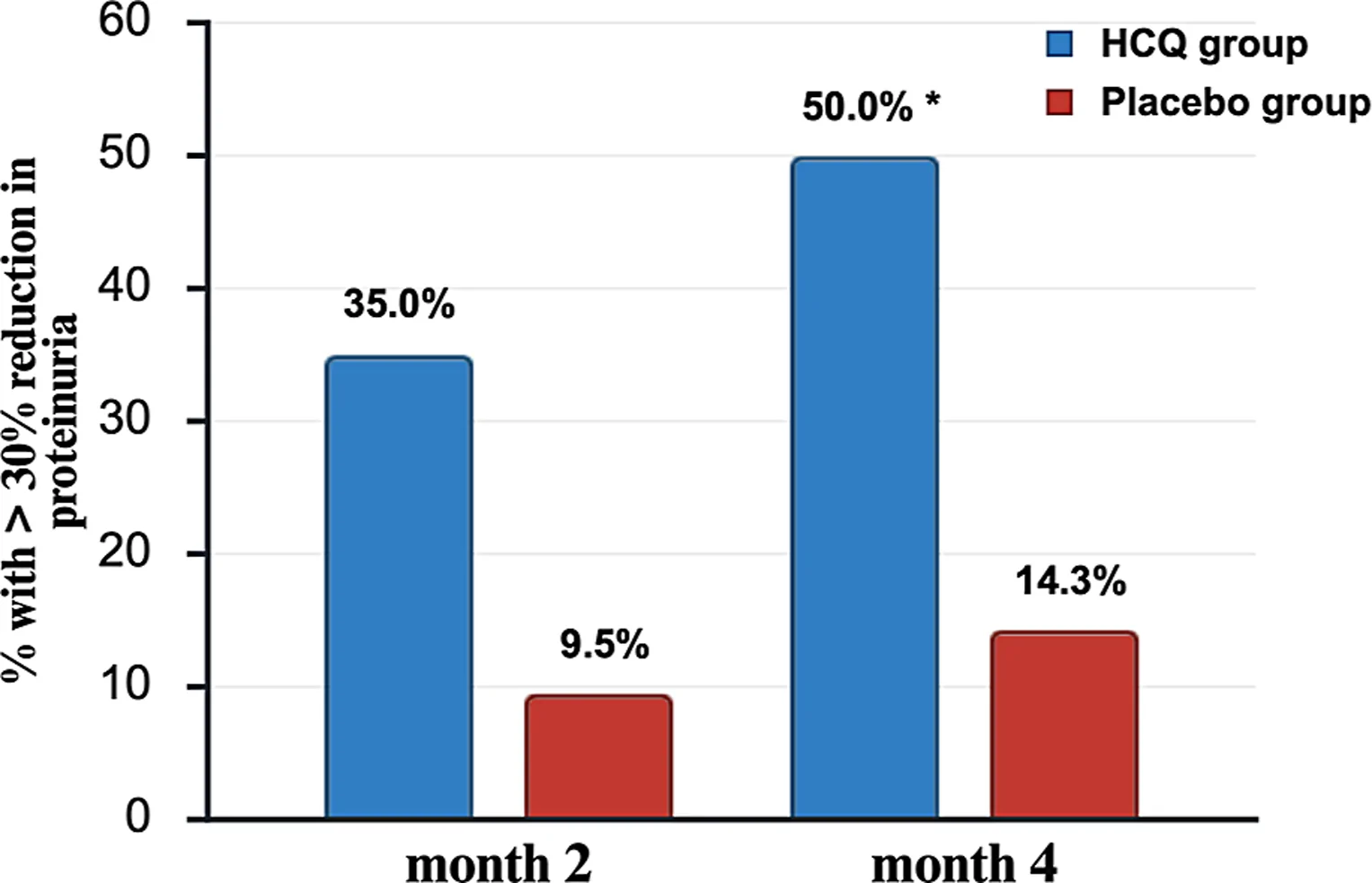
Percent with > 30% reduction in proteinuria excretion during follow-up in the HCQ group and the placebo group. *p < 0.05. Of the 41 patients who completed the month 4 visit, 10 (50%) patients in the HCQ group showed a 30% decrease in proteinuria at 4 months compared with 3 (14.3%) patients in the placebo group (p = 0.014).

### Safety and Adverse Events

No SAEs occurred in either group during the study. Adverse events are listed in Table 4. Two participants experienced adverse events in the HCQ cohort. One patient exhibited nausea and abdominal discomfort, whereas the other displayed nausea and alopecia. Both were able to tolerate the symptoms, which resolved spontaneously without dose interruption. All participants had a normal baseline 12-lead ECG with no QTc prolongation or arrhythmia; on-treatment ECG was not repeated, so cumulative cardiac effects could not be assessed within this short study.

**Table 4.** SAEs and adverse events in the HCQ group and placebo group

​	HCQ group (*n* = 21)	Placebo group (*n* = 22)	*p* value
SAEs	0	0	N/A
Adverse events			
Events, *n* (%)			
0	19 (90%)	21 (95%)	0.520
1	2 (10%)	1 (5%)	​
Event details, *n* (%)			
eGFR reduction	0 (0%)	0 (0%)	N/A
Nausea	2 (10%)	0 (0%)	0.137
Abdominal discomfort	1 (5%)	0 (0%)	0.300
Dizziness	0 (0%)	1 (5%)	0.323
Hair loss	1 (5%)	0 (0%)	0.300

*p* value by chi-square test.

## Discussion

This study evaluated the short-term antiproteinuric and anti-inflammatory effects of a 4-month course of HCQ compared with placebo in patients with stable chronic proteinuric glomerulopathy receiving optimized supportive therapy. As a single-center, double-blind, placebo-controlled proof-of-concept randomized trial, the objective was to identify a short-term biological treatment signal rather than to establish disease-specific efficacy or long-term clinical benefit. HCQ was associated with a significant reduction in proteinuria, a higher proportion of patients achieving at least a 30% reduction in proteinuria, and significant reductions in IL-6 and hsCRP after adjustment for SBP in mixed-effectsML regression analyses. These findings were supported by prespecified sensitivity analyses using log-transformed mixed-effects models. No significant difference in eGFR was observed between treatment groups over the 4-month follow-up, and HCQ was generally well tolerated during the study period.

Previous randomized trials have demonstrated antiproteinuric effects of HCQ in selected glomerular diseases, particularly IgA nephropathy and membranous nephropathy, where HCQ enhanced proteinuria remission and reduced urinary protein excretion when added to standard therapy [5, 12]. Unlike those studies, our trial intentionally enrolled a broader population of stable chronic proteinuric glomerulopathies, including IgA nephropathy, DN, and other chronic glomerular diseases, to evaluate whether HCQ might exert beneficial effects across a shared clinical phenotype characterized by persistent proteinuria and chronic inflammation. Consequently, the observed treatment effect should not be interpreted as evidence of uniform efficacy across individual glomerular diseases but rather as preliminary evidence supporting further disease-specific investigation.

Persistent proteinuria is a major predictor of CKD progression regardless of the underlying glomerular disease, and reduction in proteinuria is widely accepted as an important surrogate marker of treatment response in clinical trials [13]. The persistence of the antiproteinuric effect after adjustment for SBP and its consistency across sensitivity analyses strengthen the internal validity of our findings. Nevertheless, because of the modest sample size, biological heterogeneity of the study population, and relatively short follow-up, these results should be interpreted as preliminary and hypothesis-generating.

HCQ enhanced the remission frequency of proteinuria and reduced urine protein excretion compared with RAAS inhibitor therapy alone. In conjunction with prior trials, our data are consistent with the efficacy and safety of HCQ in individuals with chronic glomerulopathy. HCQ is extensively utilized in conditions related to immunological disorders such as systemic lupus erythematosus, rheumatoid arthritis, and Sjögren's syndrome. HCQ has demonstrated efficacy in enhancing proteinuria remission rates [14], reducing the risk of CKD [15], diminishing the necessity for recurrent kidney biopsies [16], and facilitating prolonged renal remission in LN [17]. HCQ is thought to exert pleiotropic immunomodulatory actions, such as reducing inflammatory cell activation, lowering autoantigen presentation, and obstructing Toll-like receptors and synthesis of proinflammatory cytokines [18, 19].

Experimental studies suggest that HCQ may improve endothelial nitric oxide bioavailability through modulation of intracellular iron metabolism, thereby enhancing endothelial function and vascular homeostasis [20–22]. However, we observed no significant effect on blood pressure, likely because all participants were already receiving optimized RAAS inhibition with well-controlled blood pressure before enrollment.

Early pharmacokinetic studies suggest that suppression of inflammatory cytokines may correlate with therapeutic whole-blood HCQ concentrations [23]. In this study, HCQ dosing was adjusted according to kidney function to minimize drug accumulation, but whole-blood HCQ concentrations were not measured because these assays were unavailable at our institution. Consequently, we were unable to examine exposure-response relationships or pharmacodynamic parameters such as the association between HCQ concentrations and IL-6 suppression. Future studies incorporating therapeutic drug monitoring will be important to optimize dosing strategies and better define the pharmacokinetic-pharmacodynamic relationship of HCQ in chronic glomerular disease.

No significant between-group difference in eGFR was observed during follow-up. Given the relatively slow progression of chronic glomerular disease and the 4-month study duration, the trial was not expected to detect meaningful differences in kidney function trajectory or long-term renal outcomes.

Over the 4-month treatment period, HCQ was generally well tolerated, and no SAEs were observed. However, the limited number of treated participants and short duration of follow-up preclude definitive conclusions regarding long-term safety or uncommon adverse events. Furthermore, HCQ dosing was adjusted according to eGFR rather than actual body weight, although current ophthalmological guidelines recommend maintaining the daily dose below approximately 5 mg/kg of actual body weight to minimize retinal toxicity. Future studies should incorporate body weight, therapeutic drug monitoring, serial electrocardiographic assessment, and longer follow-up to better characterize the long-term safety profile of HCQ in patients with chronic glomerular disease.

This study has several limitations. First, as an early-phase proof-of-concept trial with a relatively small sample size, the findings should be considered exploratory and hypothesis-generating rather than definitive, and the influence of random variation or regression to the mean cannot be completely excluded. Second, the study included biologically heterogeneous diseases (IgA nephropathy, DN, and other chronic glomerulopathies), and not all participants had biopsy-confirmed disease, limiting disease-specific inference and assessment of histologic activity or chronicity. Third, although background therapy was standardized with stable optimized RAAS blockade and no initiation of additional kidney-protective therapies during follow-up, residual variation in supportive care may have influenced proteinuria. Fourth, the 4-month follow-up was designed to evaluate short-term changes in proteinuria and inflammatory biomarkers and was not intended to assess durability of response, kidney function decline, long-term safety, or clinical outcomes. Finally, the biomarker and subgroup analyses were exploratory, underpowered, and not adjusted for multiple comparisons and therefore should be interpreted cautiously. The single-center design and highly selected study population also limit generalizability. Future multicenter randomized trials with larger, biopsy-proven, disease-specific cohorts and longer follow-up are required to confirm these preliminary findings and determine the long-term clinical benefits of HCQ. Such trials should also standardize background antiproteinuric therapy – including protocol-defined RAAS-inhibitor dosing and prespecified, uniform use of SGLT2 inhibitors and other guideline-directed antiproteinuric agents – so that the independent impact of HCQ on proteinuria and inflammation in glomerular disease can be assessed free of the confounding effects of heterogeneous supportive care.

## Conclusion

In this proof-of-concept randomized trial, adjunctive HCQ was associated with a significant short-term reduction in proteinuria in patients with stable chronic proteinuric glomerulopathy receiving optimized supportive therapy. Reductions in IL-6 and hsCRP were also observed but should be considered exploratory. These findings support further evaluation of HCQ in larger, longer, biopsy-based, disease-specific randomized trials.

## Acknowledgments

We thank all the investigators and study teams for their participation in the study, as well as the Department of Medicine, Phramongkutklao Hospital and College of Medicine.

## Statement of Ethics

The protocol was approved by the Institutional Review Board Ethics Committee, which approved the trial at the Royal Thai Army Medical Department (IRB RTA) on April 2, 2024, with code R014h/67. This trial was registered under the Thai Clinical Trials code TCTR20250319019. All participants provided written informed consent.

## Conflict of Interest Statement

The authors declare that there are no competing interests. Dr. Bancha Satirapoj was a member of the journal’s Editorial Board at the time of submission.

## Funding Sources

This study was supported by a grant from the Department of Medicine, Phramongkutklao Hospital and College of Medicine, Bangkok, Thailand. The grant provided financial support for laboratory investigations and research medications used in this study. The funding source had no role in the study design, execution, data analysis, manuscript preparation, or decision to publish.

## Author Contributions

T.S., P.T., O.S., and B.S. contributed to the concept and design of the study. T.S. and B.S. contributed to data acquisition, analysis, and interpretation. T.S. wrote the first draft of the manuscript. All authors approved the final version of the manuscript.

## Data Availability

The data that support the findings of this study are not publicly available because of the risk of participant identification and the need to protect the privacy of research participants, but are available from the corresponding author upon reasonable request.
